# Identification of New Respiratory Viruses in the New Millennium

**DOI:** 10.3390/v7030996

**Published:** 2015-03-06

**Authors:** Michael Berry, Junaid Gamieldien, Burtram C. Fielding

**Affiliations:** 1 South African National Bioinformatics Institute, University of the Western Cape, Western Cape 7535, South Africa; E-Mails: michael@sanbi.ac.za (M.B.); junaid@sanbi.ac.za (J.G.); 2 Molecular Biology and Virology Laboratory, Department of Medical BioSciences, Faculty of Natural Sciences, University of the Western Cape, Western Cape 7535, South Africa

**Keywords:** respiratory viruses, human coronaviruses, hMPV, bocavirus, SARS-CoV, MERS.

## Abstract

The rapid advancement of molecular tools in the past 15 years has allowed for the retrospective discovery of several new respiratory viruses as well as the characterization of novel emergent strains. The inability to characterize the etiological origins of respiratory conditions, particularly in children, led several researchers to pursue the discovery of the underlying etiology of disease. In 2001, this led to the discovery of human metapneumovirus (hMPV) and soon following that the outbreak of Severe Acute Respiratory Syndrome coronavirus (SARS-CoV) promoted an increased interest in coronavirology and the latter discovery of human coronavirus (HCoV) NL63 and HCoV-HKU1. Human bocavirus, with its four separate lineages, discovered in 2005, has been linked to acute respiratory tract infections and gastrointestinal complications. Middle East Respiratory Syndrome coronavirus (MERS-CoV) represents the most recent outbreak of a completely novel respiratory virus, which occurred in Saudi Arabia in 2012 and presents a significant threat to human health. This review will detail the most current clinical and epidemiological findings to all respiratory viruses discovered since 2001.

## 1. Introduction

Viral infections of the upper and lower respiratory tract are among the most common illness in humans. Children and infants bear the major burden of infection, typically presenting with 5 to 6 episodes annually [1]. These infections are often associated with significant patient morbidity and related mortality. For this reason, URTIs and LRTIs represents the leading cause of death in children younger than five years of age worldwide [2,3]; this accounts for approximately 4 million deaths annually [4]. Acute respiratory tract disease is the leading cause of hospitalization in children and febrile episodes in infants younger than three months of age [5,6].

Bacteria only represent approximately 10% of all upper respiratory tract infections with the subsequent 90% of infections caused by respiratory viruses [7]. Despite the viral aetiological origin of most respiratory infections, antibiotics are often prescribed in the treatment of such diseases [8], exacerbating antibiotic abuse. The morbidity and fiscal implications associated with respiratory infections are significant, with approximately 500 million cases reported in the United States alone each year with subsequent direct and indirect costs to the US economy estimated at $40 billion annually [2]. The burden of respiratory tract infections is increased in patients with chronic comorbidities or clinical risk factors including asthma [9], chronic obstructive pulmonary disease (COPD) [10], young, elderly [11] and immunocompromised [12,13].

The viruses primarily associated with upper respiratory tract infections commonly include rhinoviruses, enteroviruses, adenoviruses, parainfluenza viruses (PIV), influenza viruses, respiratory syncytial viruses (RSV) and coronaviruses [3,8,14,15]. In recent years six new human respiratory viruses have been reported including human metapneumovirus (hMPV) [16], bocavirus and four new human coronaviruses including Severe Acute Respiratory Syndrome coronavirus (SARS-CoV), human coronavirus NL63 (HCoV-NL63), HCoV-HKU1 and Middle East Respiratory Syndrome coronavirus (MERS-CoV). This review will detail these newly discovered and emerging respiratory viruses.

## 2. Human Coronaviruses

Coronaviruses affect a diverse group of animal hosts, and cause a plethora of diseases in animals including progressive peritonitis, acute and chronic hepatitis, gastroenteritis, nephritis, and encephalitis [17]. In humans coronavirus infection results in respiratory tract complications with varying degree of severity and have been associated with gastroenteritis. Four human coronaviruses (HCoV-229E, HCoV-OC43, HCoV-NL63 and HCoV-HKU1) are endemic in the human population and are mainly associated with mild, self-limiting respiratory illnesses. Another two human coronaviruses, namely SARS-CoV and MERS-CoV cause severe respiratory syndromes and present a significant threat with their high fatality rates.

The first human coronaviruses were identified in the 1960s by Tyrell and Bynoe who passaged a virus, named B814, in human embryonic tracheal organ cultures. When this virus was inoculated intranasally into human volunteers, common cold-like symptoms were produced [18,19]. Hamre and Procknow (1966) later isolated a virus, which they were able to grow in tissue culture, from subjects presenting with symptoms of the common cold. The virus was later named human coronavirus 229E (HCoV-229E) [20].

All of these viruses, including B814 and HCoV-229E, were ether-sensitive, indicating the presence of a lipid covering. McIntosh *et al.* (1967) later recovered several ether-sensitive viruses from human respiratory tracts, which were grown in organ culture. These viruses were termed “OC” viruses to indicate passage in organ culture. Under electron microscopy all of these viruses had morphologies similar to several other animal viruses previously identified, namely IBV, MHV and TGEV [21]. These viruses were later classified as coronaviruses, which was accepted as a new genus in 1975. Unfortunately, many of the clinical samples collected and stored in the 1960s, which were positive for coronavirus-like particles, were subsequently lost. Therefore, until the identification of SARS-CoV in 2003, the study of human coronaviruses was restricted to research of HCoV-229E and HCoV-OC43 [17] despite the clear evidence of further coronavirus strains [22,23].

### 2.1. Severe Acute Respiratory Syndrome Coronavirus: Emergence of a Novel Coronavirus

An outbreak of atypical pneumonia was first reported to the WHO in February 2003. By the 15th of March the disease was named Severe Acute Respiratory Syndrome (SARS) [24,25,26] and by the 27th of March the causative agent was identified as a completely novel coronavirus, termed SARS-CoV [27,28,29]. From November 2002 to July 2003 a total of 8098 patients, in 25 countries, were affected by the atypical pneumonia which resulted in 774 deaths globally. The mortality rates were drastically increased in certain population [30] and age [31] groups to as high as 40%–55%. Further local outbreaks were reported in Singapore, Taiwan and Beijing from accidental laboratory exposure and animal to human transmission in Guangzhou in late 2003 and early 2004 [32]. This was directly related to the lifting of a ban on serving palm civets in wet markets and restaurants, which was implemented during the SARS outbreak [33].

No antiviral agents were available during the outbreak to combat infection with SARS-CoV [34] and supportive care and administration of antibiotics to combat secondary bacterial infection was the primary treatment regimen [35]. With this it is clear that the control of the SARS-CoV outbreak is thanks, almost entirely, to a highly effective global public health response [36], where intense contact tracing and quarantine of suspected and infected patients were key in the control of the SARS outbreak [35]. SARS-CoV is no longer in human circulation but the discovery of SARS-like-CoV in bats and other mammals does present a potential threat of re-emergence [37].

The outbreak of SARS-CoV was most-likely linked to a zoonotic event in the live animal markets of China. Himalayan palm civets and raccoon dogs were the first animals identified to carry a SARS-like-CoV with 99.8% nucleotide homology to human SARS-CoV [38]. Further studies of wild animals in the areas around Hong Kong identified a further SARS-like-CoV in Chinese horseshoe bats (genus *Rhinolophus*), which shared a sequence identity of 87%–92% with human SARS-CoV [39,40]. Horseshoe bats therefore appear to be the natural reservoir of the ancestral SARS-CoV, with civets providing the intermediate amplification host with subsequent spread to animal handlers in the wet markets of Guangzhou. Under positive selection pressures within the human host SARS-CoV became readily transmissible and allowed for fast and effective spread from human-to-human with subsequent international dissemination by the index case in Hong Kong [41]. Analysis of genomic sequences from viral isolates obtained in the early, mid and late stages of the epidemic clearly indicate positive and purifying selective pressures followed by stabilization [33]. Seroepidemiological studies have shown that 40% of wild animal traders and 20% of people responsible for the slaughtering of animals in the region where human SARS was thought to originate, were seropositive for SARS-CoV, although all cases were asymptomatic. This indicates that these people were previously exposed to a SARS-like-CoV, which resulted in asymptomatic infection [38].

SARS presents as an atypical pneumonia [42,43], with pneumocytes being the primary target of infection. Infection results in haemorrhagic inflammation in most pulmonary alveoli with alveolar thickening, diffuse alveolar damage, desquamation of pneumocytes, formation of hyaline membranes and multinucleated pneumocytes with capillary engorgement and microthrombosis [44]. Approximately 60% of patients deteriorated in the second week of infection, presenting with persistent fever, dyspnoea and oxygen desaturation [29]. Approximately 20%–30% of patients were subsequently admitted to intensive care, where mechanical ventilation was necessary [45]. A surprising finding with the SARS outbreak was that it was not as great a threat to infants and children [33,46]. Clinical presentation was less severe in infants and no children aged between 1 and 12 required intensive care or mechanical ventilation [47,48]. This is in sharp contrast to the age related burden of other respiratory infections and the underlying biological mechanism remains unclear [49].

A family of viruses that were previously understood to cause mild, self-limiting upper respiratory tract infections was showcased by the SARS-CoV outbreak to be a significant threat to global public health. The economic losses brought on by the SARS pandemic was estimated to be in the region 40 billion dollars [50] with Hong Kong bearing a significant proportion of the losses. Tourism, entertainment and restaurant industries in the area recorded up to 80% loss in business. The pressure on healthcare facilities in affected areas was substantially exacerbated by the spread to healthcare workers. Several hospitals were forced to close to new admissions as large numbers of staff became infected with SARS-CoV. To view this as an isolated incidence would be naïve and the potential for the emergence and re-emergence of new and existing infectious agents poses a probable risk. Understanding the SARS-CoV outbreak has provided immense knowledge and an excellent model to replicate in the event of further outbreaks of communicable diseases.

### 2.2. Human Coronavirus NL63 and HKU1: Discovery of Existing Coronaviruses

#### 2.2.1. Human Coronavirus NL63

In 2003 a 7 month old child presenting with bronchiolitis and conjunctivitis was screened for several respiratory viruses to identify the causative agent, with all diagnostics yielding negative results. The group led by Lia van der Hoek then used a modified cDNA amplified restriction fragment-length polymorphism (cDNA-AFLP) technique (Virus-Discovery-cDNA-AFLP or VIDISCA), to identify the causative agent. Briefly, the technique utilizes reverse transcription-PCR of viral RNA with subsequent restriction digest of the cDNA using frequently cutting restriction enzymes. Since the restriction sites are selected and therefore known, the resultant “sticky ends” can be ligated into anchors for amplification and sequencing with specific primers. The results showed highest sequence similarities with known coronaviruses, but with significant sequence divergence indicating the discovery of a new coronavirus species, later named human coronavirus NL63 [51].

At about the same time, two other independent groups identified essentially the same virus [52,53]. Shortly after the van der Hoek paper [51], a novel coronavirus that replicated efficiently in tertiary monkey kidney and Vero cells, was retrospectively isolated from a nose swab sample collected in 1988 from an 8 month-old boy presenting with pneumonia. This virus was reported to be similar to HCoV-NL63 and named HCoV-NL [52]. In 2005, Esper *et al.* (2005) also reported the identification of a novel coronavirus isolated in New Haven, Connecticut, which was named HCoV-NH. This novel virus was identified by PCR which was adapted to amplify a conserved region within the replicase 1a or *pol* gene [53]. Subsequent sequence analysis showed that these three viruses were essentially the same virus or variants thereof [53,54].

It is obvious that HCoV-NL63 has been circulating in the human population since before 1988 [52]. In fact, molecular clock analyses have shown that HCoV-NL63 and HCoV-229E diverged from a most recent common ancestor, in a zoonotic event, approximately 563 to 822 years ago [55]. HCoV-NL63 later diverged into two lineages with subsequent recombination of the two lineages during co-infection. This frequent recombination has given HCoV-NL63 a mosaic structured genome with multiple recombination sites [56].

#### 2.2.2. Human Coronavirus HKU1

A 71 year old male patient from China was admitted to hospital with pneumonia in January 2004. Viral cultures, RT-PCR and direct antigen detection from nasopharyngeal aspirate were all negative for respiratory viruses. A pan-coronavirus RT-PCR targeting a conserved region of the *pol* gene confirmed the presence of a coronavirus however attempts to culture the causative agent were all unsuccessful. Sequencing the gene segment amplified by the pan-coronavirus assay indicated a high homology to other viruses of the βCoV genus including HCoV-OC43 but of novel origin. The human coronavirus, termed HCoV-HKU1, was later isolated from another female patient. Efforts to culture the virus had posed complicated and the complete genome was isolated, amplified and sequenced directly from RNA extracted from a nasopharyngeal aspirate [57]. Successful propagation of the virus was achieved recently in human ciliated airway epithelial cells [58] but culturing of HCoV-HKU1 still remains a daunting task. The presence of the HE gene further characterizes HCoV-HKU1 as belonging to the βCoV genus.

Since the discovery of HCoV-HKU1, its prevalence has shown a global distribution and retrospective analysis on stored nasopharyngeal aspirates have confirmed its existence since 1995 [59]; however, phylogenetic analysis suggests a much earlier divergence.

### 2.3. MERS-CoV: 10 Years on from SARS

June 2012 saw the most recent emergence of a completely novel strain of human coronavirus. A sputum sample was collected from a 60 year old male patient presenting with severe respiratory disease in the Dr. Soliman Fakeeh Hospital in Jeddah, Saudi Arabia. Viral assays frequently used could not identify any aetiological agent responsible for the disease. The sputum sample was sent to Dr. Ron Fouchier at the Erasmus Medical College in Rotterdam, Netherlands, where the virus was identified as a novel coronavirus, provisionally termed HCoV-EMC (human coronavirus Erasmus Medical College). The patient later succumbed to the disease with acute pneumonia and subsequent renal failure [60]. A retrospective study further traced the virus back to April 2012 where an outbreak of pneumonia, resulting in two fatalities, occurred in health care workers in an intensive care unit in Zarqa, Jordan [61].

Since the initial discovery, several new isolates were identified and described in scientific literature, databases and media under various names. This provoked the convening of the Coronavirus Study Group to introduce a naming convention and avoid confusion within the research field, health care authorities, governments and general public. The name Middle East Respiratory Syndrome coronavirus (MERS-CoV) was coined and has been widely accepted by the discoverers of the virus and pioneers of the field, the WHO and Saudi Ministry of Health [61].

Primary infections, to which all cases were linked directly or indirectly, occurred in Middle Eastern countries including Saudi Arabia, Qatar, Jordan, Oman and United Arab Emirates and subsequently spread to United Kingdom, Tunisia, France, Italy and Germany with Egypt and the United States recently reporting their first laboratory confirmed cases [62,63]. The potential of widespread pandemic outbreak is thought to be low as human-to-human transmission is largely inefficient [64] and only reported in close, sustained contact [65] such as within families [66], healthcare workers [67] and as nosocomial transmission [68], especially if the patient represents with comorbidities. There has been no evidence to suggest sustained community transmission [62]. The ever increasing case numbers and countries affected by the disease however suggests MERS-CoV does represent potential for widespread, global outbreak [69].

From the first reported case in June 2012, until 7 February 2014, a total of 182 cases had been reported with 79 fatalities. According to the latest figures available from the WHO, as of 11 June 2014, the virus had resulted in a total of 699 laboratory confirmed cases with a total of 209 fatalities [70]. These statistics indicate that within a 4 month period case numbers had more than trebled suggesting the virus is far from controlled. The fatality rate of 30% is also substantially increased in patients with comorbidities, with a large number of reported cases being identified in immunocompromised patients or those with underlying disease [68,71] and a severe threat of nosocomial transmission has been demonstrated [72]. Clinical presentation is similar to SARS and includes a spectrum of respiratory diseases with the most common symptoms including cough, fever and gastrointestinal symptoms [69] before progressing to pneumonia [68]. Acute respiratory distress syndrome (ARDS), renal failure, pericarditis and disseminated intravascular coagulation have also been reported [69]. The potential of widespread pandemic outbreak is thought to be low as human-to-human transmission is largely inefficient [64] and only reported in close, sustained contact [65] such as within families [66], healthcare workers [67] and as nosocomial transmission [68], especially if the patient presents with comorbidities. There has been no evidence to suggest sustained community transmission [62]. The ever increasing case numbers and countries affected by the disease however suggests that MERS-CoV does represent potential for widespread, global outbreak [69].

The inefficient spread between infected patients strongly suggests zoonotic transmission, however over two years on from the initial discovery of MERS-CoV it is still unclear where it originated from and how it infects humans. During the outbreak of SARS-CoV the identification of the wet markets of China as the probable source greatly contributed to the control of the disease [38]. This highlights the importance in identifying the origins of disease outbreak. Phylogenetic analysis places it in the same genus as SARS-CoV but within a new lineage, 2c. The closest detectable relatives suggests origins from bat coronavirus species with relative high sequence homology between Bat-CoV HKU4 and HKU5 in China [73], VM314 in the Netherlands [74] and a recently discovered isolate from South Africa [75]. Assuming that bats also form the immediate source is improbable and as with SARS-CoV an intermediate animal host species for transmission to humans is of greater likelihood [61]. High levels of neutralizing antibodies, viral RNA and infectious viruses have been discovered in dromedary camels suggesting a potential for camels to form the source for human transmission [76,77,78,79]. A recent study identified identical single nucleotide polymorphisms in a sequence of MERS-CoV isolated from an infected patient and camel, which was cared for by the patient. Phylogenetic analysis of these two sequences supported the conclusion that transmission occurred between patient and camel, however the direction of transmission could not be confirmed [80]. It has since been proven that a patient who succumbed to MERS-CoV infection obtained the virus directly from his camel herd. The direction of transmission was confirmed as camel-to-human by serological analysis [81]. These facts strongly contribute to an increasingly popular hypothesis that MERS-CoV infections in humans are directly acquired from camels, however very few patients have had reported contact with camels. Many residents of the Arabian Peninsula frequently consume unpasteurized camel milk and this has been suggested as a potential source as the virus has been detected and can remain viable for prolonged periods in milk [82,83]. The WHO, Saudi Arabia and Qatar have recently issued warnings and recommended against consuming unpasteurized camel milk and to be cautious when interacting with dromedary camels [84].

The outbreak of SARS-CoV in 2003 and MERS-CoV less than 10 years later highlights the significant threat of coronaviruses to humans and confirms that the SARS outbreak was not an isolated incident. With the ever increasing diversity of animal coronavirus species, especially within bats, the likelihood of recombination leading to future outbreaks is high and the threat of potential pandemics is real as highly pathogenic coronaviruses continue to spill over from zoonotic sources into the human population. Misdiagnosis of these outbreaks pose a further substantial threat to healthcare workers with nosocomial spread to other patients putting further pressure on an already strained healthcare system. Understanding the dynamics and molecular characteristics of human coronaviruses currently in circulation and how they emerge, infect and cause disease, we can be better prepared for future pandemics allowing for improved response, management and treatment of related conditions. Table 1 summarizes the human coronaviruses discovered since 2003.

**Table 1:** Human coronaviruses identified since 2003.

Name	Year Discovered	Emergent/Previously Circulating	Fatality Rate	Current Circulation
SARS-CoV	2003	Emergent	~10%	No
HCoV-NL63	2003	Previously circulating	low	Yes
HCoV-HKU1	2004	Previously circulating	low	Yes
MERS-CoV	2012	Emergent	~30%	Yes

### 2.4. Clinical Importance of Non-Severe Human Coronaviruses

The clinical presentation of human coronaviruses clearly follows two distinct lines of progression. SARS-CoV and MERS-CoV present with severe respiratory complications and often multisystem involvement with renal failure and enteric symptoms. The high fatality rates associated with these infections is not reflected in the remaining four human coronaviruses, HCoV-229E, HCoV-OC43, HCoV-NL63 and HCoV-HKU1. The clinical importance of SARS-CoV and MERS-CoV are evident and discussed in detail in previous sections allowing for the presentation of the involvement of the remaining non-severe human coronaviruses and there implications in human health.

#### 2.4.1. Clinical Presentation of Human Coronavirus Infection

The clinical presentation of these four non-severe human coronaviruses is largely identical and indistinguishable symptomatically, commonly presenting with rhinorrhoea, sore throat, cough and fever [85,86]. Majority of infections are associated with self-limiting upper respiratory tract disease or “the common cold” but can also present with high morbidity outcomes of the lower respiratory tract including bronchiolitis, pneumonia, [87,88,89], asthmatic exacerbations [9] acute exacerbations of chronic obstructive pulmonary disease (COPD) [10] and croup in HCoV-NL63 infected patients [90]. A report by Esper *et al.* found a correlation between HCoV-NL63 infections and Kawasaki disease [91], although other studies could not replicate the association [92,93,94]. Febrile seizures have been reported for most human coronaviruses but the significance of HCoV-HKU1 is alarming with one study indicating that 50% of patients infected with HCoV-HKU1 experience febrile seizures [9].

#### 2.4.2. Prevalence and Distribution of Human Coronaviruses

Human coronaviruses affect all age groups [85,86] but elicit more serious disease in young, elderly and immunecompromized [87,95,96], frequently resulting in hospitalization. Reports on the prevalence of human coronaviruses and their association with upper and lower respiratory tract disease vary but range between 3.3% and 16% [85,86,95,97]. Over 70% of the general public has seroconverted towards all four non-severe human coronaviruses with primary infection shown to occur in childhood [98] and reinfection occurring throughout life [95]. Given the high prevalence of respiratory infections, human coronaviruses represent a substantial disease burden which is exacerbated by the high implications of healthcare workers in coronavirus outbreaks [97].

#### 2.4.3. Human Coronaviruses Frequently Present with Co-Infection

High rates of co-infection with other respiratory viruses are commonly reported [85,86,95,99]. Viruses frequently associated with co-infection include enterovirus, rhinovirus and PIV [100] however reports of co-infection with two human coronaviruses are limited. Dijkman *et al.* recently demonstrated that HCoV-OC43 and HCoV-NL63 may elicit immunity that protects against HCoV-HKU1 and HCoV-229E, respectively [101]. Clinical progression and outcomes of disease in patients presenting with co-infection are however similar to patients presenting with mono-infection [85,95]. There is also no substantial difference in coronaviral load between co-infected and mono-infected patients. No substantial difference in disease progression in co-infected *versus* mono-infected patients has been reported and therefore understood to have little impact; however, the role in facilitation of infection of one respiratory virus by another is still speculative [95].

#### 2.4.4. Seasonal Distribution

All four human coronaviruses are endemic worldwide but the prevalence, regional distribution and pathogenicity of individual human coronaviruses is unclear and highly subjective on study conducted with parameters including population sampled, respiratory sample collected, sensitivity of diagnostic assay used, region where study was conducted and duration of the study playing a substantial role in epidemiological findings. A common finding in majority of studies conducted is the increased prevalence of human coronaviruses during winter months in temperate climates [95,97]. However even this has shown to be geographically dependent with a spring/summer predominance in subtropical and tropical climates [9,102]. Biennial outbreaks are frequently reported [85,95,99,100] for all strains of non-severe human coronaviruses.

## 3. Human Metapneumovirus (hMPV)

In 2001 a previously undiscovered virus was identified in 28 epidemiologically distinct patients in the Netherlands. Patient symptoms were similar to those infected with RSV and, several patients required hospitalization and mechanical ventilation. Viral isolates were cultured in tertiary monkey kidney (tMK) cells and cytopathic effects caused by the virus were largely identical to those caused by RSV. Electron microscopy of infected cell supernatants revealed paramyxovirus-like particles; however, RT-PCR assays to detect known paramyxoviruses were all negative. The low stringency of the assays used indicated a currently unknown, genetically distinct virus. A RAP-PCR assay was then utilized to obtain sequence information of the unknown virus and fragments amplified by the RAP-PCR allowed for further sequencing of the 3’-end of the genome. Based on the sequence homology and gene organization, the unidentified virus displayed closest homology with avian pneumovirus, but to be a tentative new member of the *Metapneumovirus* genus and the first virus in the genus to infect humans, provisionally termed human metapneumovirus (hMPV) [16].

### 3.1. Clinical Presentation of hMPV Infection

Symptomatic differentiation between hMPV and other respiratory viruses cannot be made as there is a significant overlap in clinical presentation [103,104]. The most common presentation of hMPV in children includes complications of the upper respiratory tract with rhinorrhoea, cough and fever [105]. Acute otitis media is also frequently reported [106,107] and conjunctivitis, rash, diarrhea and vomiting are reported but infrequently [103]. Bronchiolitis, pneumonia, croup and asthmatic exacerbations are the most frequently associated lower respiratory tract complications [108] and viral load is directly associated with disease severity [109]. hMPV infection in the young and elderly frequently requires hospitalization and fatalities have been reported in the elderly [110,111]. An increased morbidity in elderly patients with a delayed clearance of symptoms has been reported and is likely related to the age related impairment of the innate and adaptive immunity [103] or an over stimulated immune response leading to inflammation [112]. Elderly patients requiring hospitalization most frequently present with acute bronchitis, COPD exacerbations, pneumonia and congestive heart failure [113]. In healthy adults asymptomatic infections or cold- and flu-like symptoms are the most prevalent presentation [114].

### 3.2. Coinfections with hMPV

The pathogenesis of hMPV infection is strongly affected by bacterial coinfections with pneumococcus. One study has shown that administration of a conjugate pneumococcal vaccine is sufficient to reduce the incidence of hMPV infection of the lower respiratory tract and the incidence of clinical pneumonia in both HIV positive and negative patients [115]. These finding suggest that the incidence of hospitalizations in hMPV infections may be decreased by vaccination with a conjugate pneumococcal vaccine. Another case report of severe respiratory failure was found to be caused by coinfection with hMPV and *Streptococcus pneumonia* in a 64 year old patient [116]. Both *in vitro* and *in vivo* studies have shown that infection with hMPV facilitates adhesion of pneumococcal bacteria, which may provide an explanation for the coinfection with pneumococcal strains and hMPV [117].

Viral coinfections between hMPV and RSV have been reported, but remain a contentious issue. The typical seasonal overlap of the two viruses has been suggested to promote viral coinfection. One study reported a 10-fold increase in risk of admission to an intensive care unit in pediatric patients coinfected with RSV and hMPV and associated the dual infection as capable of augmenting severe bronchiolitis [118]. Other studies do not support this finding and further report a decreased correlation between hMPV-RSV coinfections and hospitalization and additionally lists dual infection, along with breastfeeding, as having protective effects [119].

### 3.3. Epidemiology of hMPV

Although hMPV was only discovered in 2001, it has been shown by phylogenetic analysis to have been in existence for approximately 50 years [120,121]. Soon after the discovery of hMPV, it was evident that two lineages, A and B, existed. These two lineages were further subdivided into two sublineages per lineage, A1-A2 and B1-B2 [16]. A recent report analyzing sequence divergence of the attachment and fusion surface glycoproteins indicates the presence of five sublineages, namely A1, A2a, A2b, B1 and B2 [122]. From long term retrospective studies it was evident that these lineages are not restricted to certain locations or times and that multiple lineages can exist in the same location and period [108,123]. It has also become evident that old sublineages may be replaced by new variants [103]. Disease progression or varying clinical outcomes related to different lineages of hMPV has become a contentious issue. Several studies have reported that lineage A presents with more severe clinical outcomes [124,125,126] where the same is reported for lineage B by other groups [127,128]. It has been further reported that there is no difference in disease outcomes related to the two lineages [108,129,130].

Between 7% and 19% of all cases of respiratory infections in children are caused by hMPV, in both hospitalized and outpatients [108,131,132] and has been reported to be the second most frequently identified virus in respiratory tract infections [133]. Extrapolation of consensus data suggests a total of 20 000 hospital and one million clinic visits annually in the US among children younger than 5 [131]. Children hospitalized with hMPV infections are also more likely to present with pneumonia or asthma and required longer stay in intensive care units with supplemental oxygen, when compared to other respiratory viruses [131]. Seroprevalence studies indicates that 100% of young adults are seropositive for hMPV with stable neutralizing titres, which further suggests that reinfection occurs throughout life [16], with a potential for genetic variation between clades promoting reinfection [108].

hMPV has a worldwide distribution and affects all age groups but predominantly affects young, elderly and immunocompromised patients [111], with children younger than five years of age being most susceptible to infection [134]. Children and adults with underlying or chronic conditions such as asthma, chronic lung disease, congenital heart disease, cancer or COPD are more likely to be hospitalized with hMPV infection [131]. Infection with hMPV occurs throughout the year but seasonal prevalence in late winter and spring has been observed and coincides with the peak of RSV infection [131,135,136,137].

## 4. Human Bocavirus

The first human bocavirus (hBoV) was discovered in 2005 from nasopharyngeal aspirates of 282 patients with unresolved lower respiratory tract infections in Sweden. Researchers utilized a novel technique which included steps of DNase treatment to exclude contaminating, or non-viral, nucleic acids followed by PCR amplification by nonspecific primers. The PCR-products were subsequently cloned with large-scale sequencing of the clones. Bioinformatic analysis of generated sequence data yielded the discovery of a new parvovirus with a high homology to bovine and canine minute parvoviruses. The genus name *Bocavirus* was in fact derived from the species infected by the known virus strains, namely bovine and canine. The new virus was named hBoV1 and was the first virus to be discovered by molecular virus screening [138]. Three additional species of hBoV were later discovered in 2010 and added to the genus; these were named hBoV2, hBoV3 and hBoV4 [139,140,141].

### 4.1. Clinical Significance of hBoV

HBoV1 is a respiratory pathogen affecting all regions of the globe and is associated with approximately 2%–19% of all upper and lower respiratory tract conditions [142,143,144]. HBoV1 productively infects human airway epithelium cell cultures and leads to damage of airway epithelial cells [145,146,147], which supports clinical observations that infection does result in respiratory disease. In contrast, hBoV2–4, are found in the gastrointestinal tract and hBoV2, and possibly hBoV3, are associated with gastroenteritis [139,148,149,150]. Interestingly, HBoV2 is the only enteric bocavirus to be isolated from nasopharygeal aspirates and may, therefore, also be associated with respiratory disease [151,152]. HBoV1 is detected in all age groups, but predominantly in young children between the ages of 6–24 months [143,153,154] and is rarely detected in adults [155,156,157,158,159]. Transmission and infection occurs throughout the year, but predominantly during winter and spring months [158,160,161,162]. Seroprevalence studies suggest that maternal antibodies, which provide protection, are present in infants younger than 2 months of age [163,164], after which seropositivity decreases with low levels of detection until 6–12 months. Virtually 100% of children aged 6 are seroconverted for hBoV1 and as reinfection occurs throughout life this remains into adulthood [143,163,164,165,166]. The presence of the three enteric bocaviruses does however complicate the findings of seroconversion as cross-reactivity does exist [166].

As with many respiratory viruses, clinical differentiation with hBoV1 infection is not possible by symptomatic presentation [8]. Common features of infection of the upper respiratory tract include common cold-like symptoms with cough, rhinorrhoea and acute otitis media [167]. Infection of the lower respiratory tract in children is associated with pneumonia, acute wheezing, asthmatic exacerbations and bronchiolitis [160,162,168,169,170], but life-threatening complications are rare with hBoV1 infection [143]. Although hBoV1 has been isolated from stool samples, there is no statistical evidence to associate hBoV1 with gastrointestinal disease [139]. HBoV1 has not only been found in the upper and lower respiratory tract and gastrointestinal specimens, but also in urine samples, serum, saliva, and tonsils [143]. Rather than having a role in disease pathogenesis, this viraemia and systemic spread may be a feature common to all Parvoviruses as they require proliferating host cells for replication [138].

Interestingly, hBoV appears to be more than just a respiratory or gastrointestinal virus. In a recent study, hBoV was identified in 18.3% of lung (*n =* 11/60) and 20.5% of colorectal (*n =* 9/44) tumors screened. Unfortunately, the study did not investigate whether the hBoV genomes were in fact incorporated into the host genome as reported for other known Parvoviruses. Therefore, based on their observations as well as previous studies on other parvoviruses, the authors speculate that hBoV could contribute to the development of some lung and colorectal tumors. However, they do also acknowledge that these tumors could simply be providing an optimal environment for hBoV replication and more conclusive studies are required to resolve this issue [171].

### 4.2. Coinfection with hBoV1

HBoV1 has been associated with a prolonged period of persistence in the mucosa of the respiratory tract. This prolonged presence has possibly led to a high frequency of coinfection found with hBoV1 infections of the both the upper and lower respiratory tract [142,157,158]. The high rate of detection of multiple respiratory viruses within up to 83% of respiratory specimens, and the presence of asymptomatic hBoV1 infections, does complicate the determination of the actual pathogenic role of HBoV [143]. High viral load is statistically associated with symptoms [172] and this may therefore be a better indication of coinfections which are related to disease severity or symptomatic presentation. It has been further suggested that patients presenting with viraemia are better candidates to assess the symptoms of disease when compared to investigations of respiratory secretions [172]. The effects and mechanisms of latency, persistence, reactivation and reinfection are however poorly understood and therefore its effects on coinfection and its contribution to active disease cannot be accurately stated [173].

## 5. Conclusions

The etiological agents of 12%–39% of lower respiratory tract infections still remain to be identified [138,158]. These results may vary significantly depending on the sensitivity of the diagnostic assay used, respiratory site sampled and even geographical location of the study but it does suggest that many uncharacterized respiratory pathogens could still remain elusive awaiting discovery. The vast improvements in molecular techniques within the past decade have however led to the discovery of four previously circulating respiratory viruses and also the rapid characterization of two completely novel viruses, namely SARS-CoV and MERS-CoV. All these viruses have varying but significant impact on human health and the potential for outbreak of completely novel, emergent respiratory viruses, seen with SARS and MERS, poses their own unique threats. Lessons learned from these viruses, and others currently in circulation, provide health care authorities and scientists with suitable expertise and knowledge to rapidly identify and combat novel respiratory viruses and as our techniques improve we will be in a position to characterize those viruses that are currently difficult to isolate and identify.
